# Secukinumab Leading to Rapid Improvement in Pyogenic Arthritis, Acne, Pyoderma Gangrenosum, and Hidradenitis Suppurativa (PAPASH) Syndrome: A Case Report and Review of Treatment Modalities for PAPASH Patients

**DOI:** 10.1155/crrh/7720064

**Published:** 2025-04-22

**Authors:** Inga N. Shevtsova, James J. Abbott, Pavel N. Shevtsov, Guiset Carvajal Bedoya, Diana C. Norton

**Affiliations:** ^1^Department of Internal Medicine, Billings Clinic, Billings, Montana, USA; ^2^Department of Dermatology, Billings Clinic, Billings, Montana, USA; ^3^Department of Rheumatology, University of Kentucky, Lexington, Kentucky, USA; ^4^Department of Rheumatology, Billings Clinic, Billings, Montana, USA

**Keywords:** neutrophilic dermatosis, PAPASH, pyoderma gangrenos, pyogenic arthritis, secukinumab, treatment modalities for PAPASHsum

## Abstract

PAPASH syndrome, a rare autoinflammatory condition characterized by pyogenic arthritis, pyoderma gangrenosum, acne, and hidradenitis suppurativa, presents significant treatment challenges due to its rarity and complex multisystem involvement. Since its initial description in 2013, only 14 cases have been documented, leading to limited treatment experience. Although IL-1 and TNF-alpha blocking agents have shown efficacy, responses vary due to genetic and pathogenetic differences, with some cases being resistant. Therefore, alongside summarizing prior treatment experiences, new treatment modalities need to be explored. This report presents the case of a 46-year-old Native American male with PAPASH syndrome who responded successfully to IL-17 inhibition with secukinumab. The patient experienced marked improvement in both dermatologic and rheumatologic symptoms, highlighting the potential role of IL-17 in the pathogenesis of PAPASH. This case suggests that IL-17 inhibition could be a promising treatment modality for PAPASH syndrome.

## 1. Introduction

PAPASH syndrome is an exceedingly rare autoinflammatory condition characterized by a tetrad of pyogenic arthritis, pyoderma gangrenosum, acne, and hidradenitis suppurativa. It was initially described in 2013 by Marzano and colleagues [1]. To date, there have been 14 cases reported [1–12], and treatment experience remains very limited. Managing the different elements of a multisystem autoinflammatory syndrome can be challenging. This can be exemplified by other autoinflammatory syndromes such as hidradenitis suppurativa, which is notoriously difficult to treat and often refractory to standard management strategies [13]. IL-1 and TNF-alpha blocking agents are usually successful in managing autoinflammatory clinical syndromes; however, clinical responses vary among patients [14], likely due to differences in genetic mutations [10] and pathogenesis. Experience in managing PAPASH syndrome is even more limited due to its rarity, especially in refractory cases. Herein is a case report of successful IL-17 inhibition treatment for PAPASH syndrome.

## 2. Case Presentation

**Figure 1:**
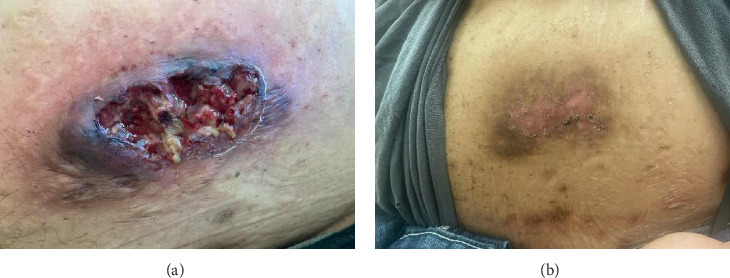
Pyoderma gangrenosum before (a) and after (b) secukinumab therapy.

**Figure 2:**
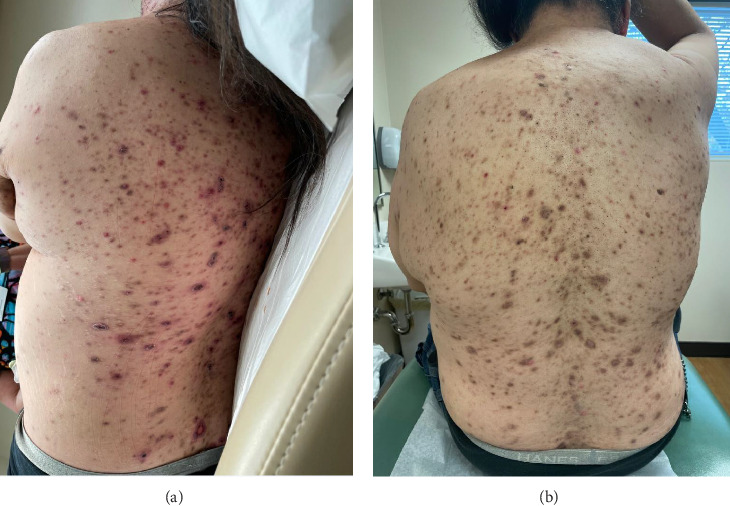
Multiple persistent acne on the back before (a) and after (b) secukinumab therapy.

A 46-year-old Native American obese male presented with a painful, bleeding ulcer on the left lower abdomen that abruptly developed one week before hospitalization (Figure 1(a)). The patient had a long history of facial and truncal nodulocystic acne (Figure 2(a)), and Hurley Stage III hidradenitis suppurativa, which includes 20 inflammatory nodules and 10 abscesses in the axillae and inguinal folds, consistent with an International Hidradenitis Suppurative Severity Score System (IHS4) of 40. The patient is obese (Body Mass Index: 32.8) and has no history of tobacco smoking or other risk factors for hidradenitis suppurativa. In addition, the patient experienced multiple episodes of inflammatory oligoarthritis, with significant joint effusion and synovial hyperplasia, affecting the wrists, ankles, and knees. No family member has similar symptoms. The patient has a history of alcohol use disorder, which severely limited his access to healthcare. Despite experiencing skin and musculoskeletal symptoms for many years, he did not undergo a comprehensive evaluation by a specialist for these conditions. His symptoms were managed with supportive and symptomatic care, such as nonsteroidal anti-inflammatory drugs, glucocorticoids, and antibiotics, through episodic visits to community primary care and urgent care facilities. In addition, the alcohol use disorder led to cardiomyopathy with reduced ejection fraction, which is currently compensated with alcohol abstinence and maximal medical therapy.

**Figure 3:**
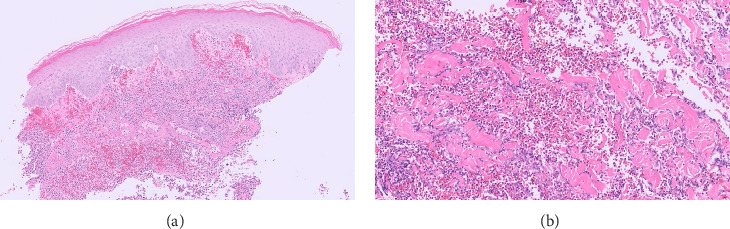
The dermis is replaced by a dense inflammatory neutrophilic infiltrate. Papillary dermal edema with extravasated red blood cells. H&E 100x magnification (a), 200x magnification (b).

Routine and immunological laboratory tests were within normal limits, except for elevated C-reactive protein (14.1 mg/dL, reference < 1 mg/dL) and erythrocyte sedimentation rate (17 mm/hr, reference < 15 mm/hr). A skin biopsy was taken from the edge of the non-healing lower abdomen ulcer, which revealed a dense neutrophilic infiltrate and mild papillary edema (Figure 3). Special staining (Periodic acid-Schiff, Gomori methenamine silver, and Gram) for microorganisms was negative. The histologic findings were compatible with a neutrophilic dermatosis.

PAPASH syndrome was diagnosed based on the clinical presentation (inflammatory polyarthritis, pyoderma gangrenosum, acne, and hidradenitis suppurativa) and histologic findings. Genetic testing for the proline–serine–threonine phosphatase-interacting protein 1 (PSTPIP1) was not performed due to limited financial resources of the patient and uncertain impact on the treatment strategy.

**Figure 4:**
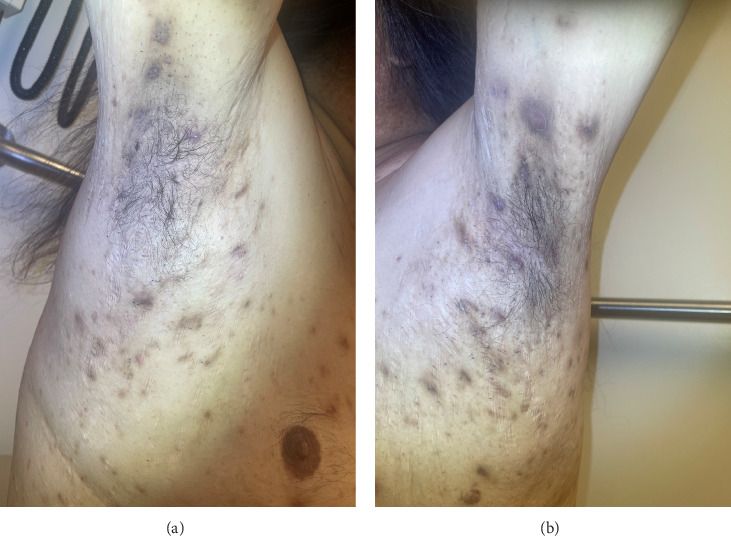
Hidradenitis suppurativa right (a) and left (b) axillary areas following secukinumab therapy.

For the treatment, secukinumab, an IL-17 inhibitor, was selected because of its favorable safety profile and availability to the patient. TNF-alpha inhibitors were contraindicated due to the patient's alcohol-induced cardiomyopathy with reduced ejection fraction. Medical insurance coverage for anakinra, an IL-1 inhibitor frequently used in many autoinflammatory conditions, was declined due to the lack of clinical trials for his condition. However, the patient qualified for secukinumab therapy coverage since it is Food and Drug Administration (FDA)-approved therapy for severe hidradenitis suppurativa. Shortly after initiating the medication, the patient demonstrated significant clinical improvement in all his dermatologic and rheumatologic symptoms. At the 4-week follow-up, the patient exhibited areas of re-epithelialization over his abdominal ulcer (Figure 1(b)), improvement in his acne (Figure 2(b)), polyarticular arthritis, and decreased inflammatory nodules in the axillae (Figure 4) and inguinal folds. At the 12-week follow-up, the patient had not developed any new wounds or skin abscesses, and his polyarticular arthritis remained in remission. The number of inflammatory nodules in the axillae and inguinal fold decreased to five, which reduced the NHS4 score to 5.

## 3. Discussion

PAPASH syndrome is a rare autoinflammatory condition characterized by aberrant neutrophilic-predominant sterile inflammation. This syndrome is part of a spectrum of neutrophilic dermatoses that includes (pyogenic arthritis, pyoderma gangrenosum, and acne (PAPA) and (pyoderma gangrenosum, acne, and hidradenitis suppurativa (PASH). Despite the identical phenotypical clinical presentation among PAPASH syndrome cases, different genetic mutations have been found, including mutations in the PSTPIP1, MEFV, NCSTN, and NLRC4 genes [10]. This potentially might explain the varied responses to different treatment modalities as well as comorbidities, such as familial Mediterranean fever and ulcerative colitis.

Due to the rarity of this syndrome, there are limited evidence-based treatment guidelines. Currently, the management of PAPASH syndrome primarily relies on extrapolation of treatment experiences from other neutrophilic dermatoses and PAPA spectrum autoinflammatory diseases.

A literature review of PAPASH syndrome case reports (Table 1) revealed 14 patients to date, ranging in age from 14 to 50 years, consisting of 6 females and 8 males. This distribution suggests a near 1:1 gender ratio among this limited number of cases. Three patients had ulcerative colitis, and one patient had familial Mediterranean fever as comorbid conditions. In addition to antibiotics, glucocorticoids, and conventional immunosuppressive therapy, the use of biologic Disease-Modifying Antirheumatic Drugs (DMARDs) was reported in 8 patients, yielding favorable clinical results. The most commonly used biologic DMARDs were TNF-alpha inhibitors (adalimumab, infliximab). IL-1 inhibition therapy (anakinra) was also utilized, including in one case that was resistant to TNF inhibition.

The exact pathogenesis of PAPASH syndrome is unknown; however, there is an overproduction of IL-1β, leading to an uncontrolled increase in proinflammatory cytokines, particularly IL-17. The latter directly recruits and activates neutrophils [15], and marked elevation of cutaneous and systemic IL-17 levels have been found in PAPASH patients [3].

Secukinumab is the first IL-17 inhibitor to be approved by the FDA for the treatment of moderate to severe hidradenitis suppurativa [16]. There are no reports of IL-17 inhibitors being used for PAPASH. However, several patients with related conditions have been treated successfully with IL-17 inhibitors, one patient with PsAPASH syndrome [17] and another with a PAPA-like autoinflammatory syndrome [18]. These syndromes clinically overlap with PAPASH and likely share a similar pathogenesis.

## 4. Conclusion

This is the first case of successful use of IL-17 inhibition in PAPASH syndrome refractory to other treatment modalities. Through this report, we highlight the role of IL-17 in the pathogenesis of PAPASH syndrome and the robust clinical response achieved through IL-17 inhibition.

## Figures and Tables

**Table 1:** PAPASH syndrome case reports and clinical response to different treatment modalities [1–12].

Author/year	#	Age sex	Comor bidity	Treatment modalities			Response/details to treatment modality
				GC	Other	bDMARD	
Marzano, 2013, 2022	1	16 F	FMF		Azithromycin Doxycycline Dapsone Colchicine	Anakinra Adalimumab	Complete response
	2	14 M		Prednisone	Rifampicin Doxycycline Clindamycin	Adalimumab Anakinra	Complete response
	3	28 F	UC		Doxycycline	Infliximab	Complete response

Faleri, 2016	1	26 M		Systemic GC	Antibacterial Isotretinoin	Anakinra	1. Antibiotics and systemic corticosteroids were ineffective. 2. Complete response to isotretinoin 30 mg daily and anakinra.

Garzorz, 2016	1	39 F			Cyclosporine	Adalimumab	1. Good response to cyclosporine with closure of PG ulcers but worsening of hidradenitis suppurativa (HS), inflammatory back pain, and polyarthritis after cyclosporine was tapered off. 2. Adalimumab initiation provides stable/partial response.

Ursani, 2016	1	44 M	UC	Prednisolone			Significant improvement of skin ulcerations and resolution of diarrhea after initiation of prednisolone (1 mg/kg), then taper down to 5 mg over 5 months.

Giovanardi 2019	1	50 F		Systemic GC	Cyclosporine	Adalimumab	Excellent clinical response to intravenous corticosteroid 1 mg/kg and adalimumab HS dosing.

Gottlieb, 2019	1	18 F					Treatment is not reported

Kawanishi K, 2021	1	26 M	UC	Prednisolone	Minocycline Dapsone Methotrexate		1. Resoluation of skin and arthritis after initiation of prednisolone (1 mg/kg), minocycline and dapsone. 2. After remission was obtained, prednisone was tapered down to 7.5 mg daily, and methotrexate was added as maintenance therapy.

Kotzerke, 2021	1	19 M			Isotretinoin		Improvement in skin lesions

Monte Serrano, 2021	1	47 M					Treatment is not reported

Al Soufi, 2023	1	47 M		Prednisone	Methotrexate		Remarkable clinical improvement in the skin ulcerations to prednisone 1 mg/kg, and methotrexate 10 mg and then prednisone was tapered down by 5 mg every week.

Ahmad, 2024	1	35 M			Colchicine Methotrexate	Infliximab Adalimumab Anakinra	1. No response to TNFi 2. Improvemnt in inflammatory polyarthritis with high-dose anakinra (200 mg) daily
	2	37 M			Methotrexate	Infliximab	Complete control arthritis, partial/complete control of skin symptoms.

## Data Availability

Data sharing is not applicable to this article as no new data were created or analyzed in this study.

## Nomenclature

bDMARD: Biologic Disease-Modifying Antirheumatic Drug
FDA: Food and Drug Administration
GC: Glucocorticoid
IL-1: Interleukin 1
IL-17: Interleukin 17
MEFV: Mediterranean Fever
NCSTN: Nicastrin
NLRC4: NLR Family CARD Domain Containing 4
PAPA: Pyogenic arthritis, pyoderma gangrenosum, and acne
PAPASH: Pyogenic arthritis, acne, pyoderma gangrenosum, and suppurative hidradenitis
PASH: Pyoderma gangrenosum, acne, hidradenitis suppurativa
PsAPASH: Psoriatic arthritis, pyoderma gangrenosum, acne, and suppurative hidradenitis
PSTPIP1 gene: Proline–serine–threonine phosphatase-interacting protein 1 gene
TNF-alpha: Tumor necrosis factor alpha
UC: Ulcerative colitis
